# A Cheerful Home

**Published:** 1873-01

**Authors:** 


### A CHEERFUL HOME.

Let there be a blazing fire on one of Dixon's hearths; let the lamps be brightly burning after nightfall; let cheering words and courteous speeches, and laughing eyes and beaming countenances be the order of the night, as soon as "Father" comes home from the work or business of the day. Let affection and self-abnegation rule the house the night, the year, the lifetime. Let "Mother" be always consulted, looked up to, respected, loved—loved so dearly, that no word or act or look shall be other than what will make her more loving, more happy—thus adding happiness to the household and setting a noble example to all.

